# How accurately do adult patients report their absence seizures?

**DOI:** 10.1002/epi4.12689

**Published:** 2023-03-28

**Authors:** Joao Pizarro, Suzanne O'Sullivan, Matthew C. Walker

**Affiliations:** ^1^ Department of Clinical and Experimental Epilepsy UCL Queen Square Institute of Neurology London UK; ^2^ Chalfont Centre for Epilepsy Buckinghamshire Chalfont St Peter UK

**Keywords:** absence seizures, antiseizure medication, genetic generalized epilepsy, self‐report, video‐EEG telemetry

## Abstract

We depend upon self‐reporting to determine seizure frequency for epilepsy management decisions, but people often misreport their seizures. Here, we determined misreporting rates in adults with absence seizures, undergoing inpatient video‐EEG telemetry (VET) or outpatient ambulatory electroencephalography (aEEG). Under‐reporting rates were based on VET data, where behavior could be assessed, whilst over‐reporting was assessed using both VET and aEEG. Forty‐two patients (31 female and 11 males, median age 28.5 years) and 759 reported absence seizures were included in this study. Overall, only 24% of the 759 reported seizures had an associated EEG correlate, indicating a high over‐reporting rate, which occurred in 57% of patients. Age, sex, time of epilepsy, VET versus aEEG, epilepsy syndrome, or medication were not significant predictors of over‐reporting. In the VET group in which we could assess both over‐ and under‐reporting (22 patients), only 2 patients correctly reported their seizures, and patients were predominantly over‐reporters or under‐reporters, not both. Only 26% of 423 absence seizures were reported. Use of zonisamide or valproate was associated with under‐reporting, possibly through an impact on attention. These findings indicate that self‐reported absence seizures are a poor measure to use for treatment decisions due to both over‐ and under‐reporting.

## 1 INTRODUCTION

We largely depend upon self‐reporting to determine seizure frequency for epilepsy management decisions. In both inpatient and outpatient settings, patients are given diary sheets that reliably record the seizures people recall^1^ but do not necessarily reflect the seizures that people have.^2^, ^3^, ^4^, ^5^, ^6^ Most of these studies have determined misreporting of focal seizures. However, we were concerned that there was an even higher rate of misreporting in adults with typical absence seizures (AS), either through under‐reporting (having AS but not reporting them) or over‐reporting (reporting AS without associated EEG correlate). Under‐reporting by parents of children with absence seizures has been described previously,^7^, ^8^ and more recently, under‐ and over‐reporting of absences and myoclonus in adults using home VET has been reported,^8^ but, in that study, absence seizures during runs of spike–wave were defined using behavioral or EEG criteria due to problems inherent to home VET. Here, we used both outpatient ambulatory EEG (aEEG) and inpatient VET to determine over‐reporting but only inpatient VET to determine under‐reporting of behaviorally defined absence seizures.

## 2 METHODS

### 2.1 Patients

All inpatient and outpatient video‐EEG telemetry (VET) and ambulatory electroencephalogram (aEEG) reports, from January 2015 until May 2021, from the Chalfont Centre for Epilepsy archive, were retrospectively analyzed together with patient diary sheets.

Only patients with an established diagnosis of genetic (idiopathic) generalized epilepsy (GGE) and AS or with an EEG report indicative of GGE and AS were included. Patients, who reported AS without specifying the time or in an ill‐defined period, so that correlation with EEG was not possible, were excluded. Where patients were unable to record seizures (eg, those with significant learning disabilities), reports of the caregivers and/or relatives were used instead. The number of patients who reported AS were then compared with the number of EEG‐identified AS. Only VET data were used to determine under‐reporting, as it provides sufficient information for unreported seizure detection (correlation between video and EEG recording), whilst it is not possible to determine a clinical event with ambulatory EEG alone. Only events during VET with a behavioral correlate were included *as* absence seizures for the under‐reporting analysis. Over‐reporting was determined by the reporting of an event without an associated EEG correlate, and thus, both VET data and data from ambulatory EEG could be used. Antiseizure medications were also assessed and grouped into categories according to putative target (Table S1). This study was approved by the National Hospital for Neurology and Neurosurgery Research Ethics Committee as a service evaluation; individual consent was, therefore, not required.

## 3 STATISTICAL METHODS

We performed logistical regression analyses for over‐reporting and under‐reporting separately. The analysis of over‐reporting was performed on all the data, and the under‐reporting was performed on just those who had VET where the presence of a seizure could be determined by video or interaction with staff (see above). We used age, VET/ambulatory EEG, sex, epilepsy syndrome, medication type, and length of time of epilepsy as factors, and since this was an exploratory study, we used forward stepwise regression using a probability of stepwise entry of 0.05. To determine the relationship between two binary variables, we used the phi coefficient. *P* < 0.05 was our criterion for a significant result. Statistical analysis was performed in SPSS v28.

## 4 RESULTS

A total of 42 patients (31 female and 11 males, median age 28.5 years ranging from 18 to 73 years old) were included in this study. All had genetic generalized epilepsy with typical absence seizures. Twenty‐one had juvenile absence epilepsy, 10 had juvenile myoclonic epilepsy, five had childhood absence epilepsy, three had epilepsy with eyelid myoclonia, and two had unclassified genetic generalized epilepsy. People were on a median of two antiseizure medications. Two people were on no antiseizure medication, 7 were on monotherapy, and 33 were on polytherapy. Ten (24%) were on medication usually contraindicated in absence epilepsy (carbamazepine, oxcarbazepine, eslicarbazepine, or pregabalin), used mostly here to control refractory tonic–clonic seizures.

**FIGURE 1:**
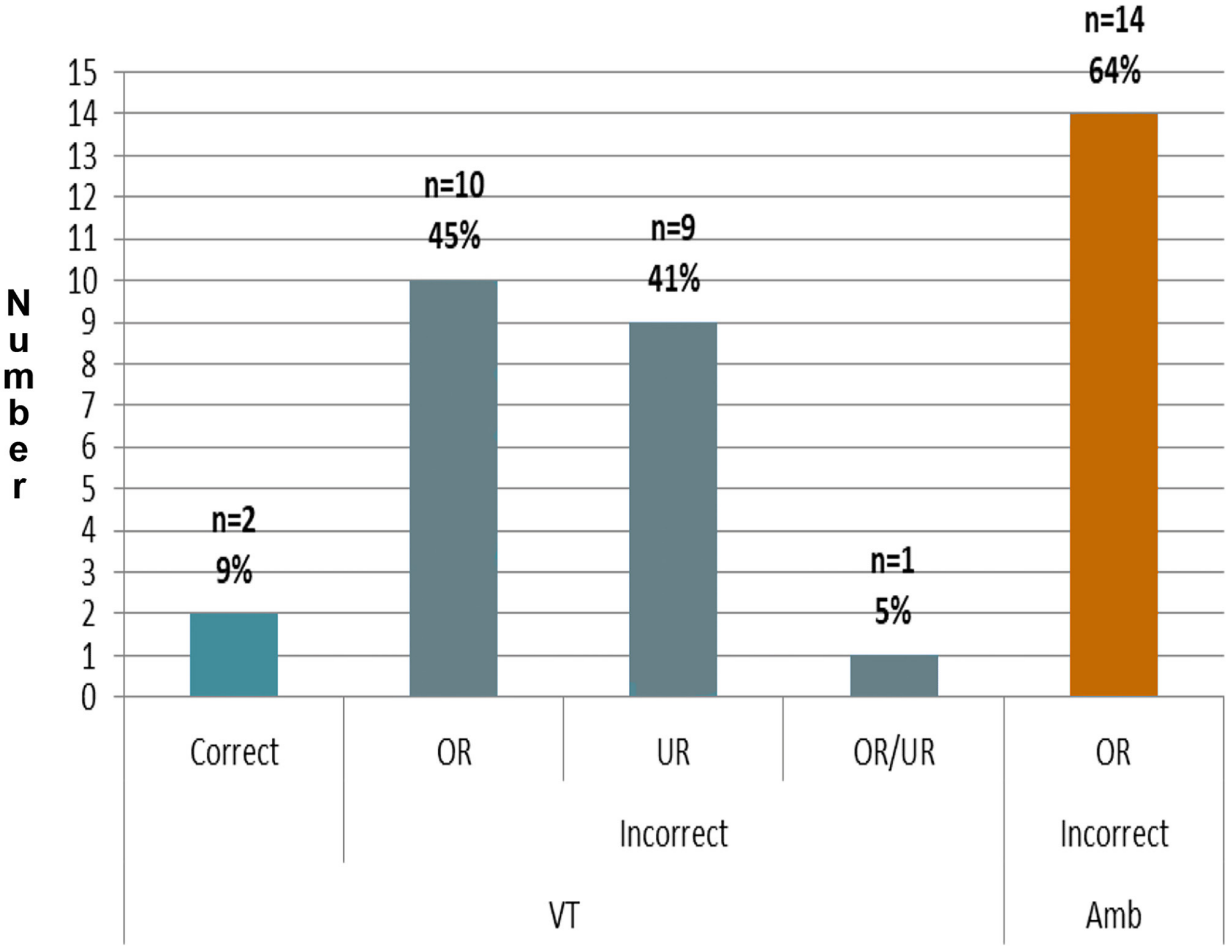
Number of patients who over‐reported (OR), under‐reported (UR), or did both (OR/UR) and correctly reported for the VT group and OR for the ambulatory group

The cohort was divided into (1) those in whom we could adequately assess under‐reporting (the video‐EEG telemetry group) as, in this group, we could match generalized spike/wave or polyspike/wave EEG activity to a clinical event and (2) those in whom we could assess over‐reporting (ambulatory and video‐EEG telemetry group) when a reported event has no associated EEG correlate. Twenty‐four of 42 patients (57%) over‐reported their absence seizures. Twenty females (60%) and 4 males (36%) over‐reported their seizures (Figure 1). In total 759 absence seizures were reported of which only 24% were absence seizures (ie, associated with an EEG correlate). In the logistic regression analysis, age, sex, length of epilepsy, VT/ambulatory EEG, epilepsy syndrome, or antiseizure medication type were not significant predictors of over‐reporting.

In the 22 patients in the under‐reporting assessment (VET) group, 10 patients (45%) over‐reported, 9 (41%) under‐reported, 1 (5%) both over‐ and under‐reported, and 2 (9%) correctly reported their seizures. 40% of females and 43% of males under‐reported their seizures (Figure 1). In this group, 473 absence seizures were recorded, of which 26% were reported. Four people with absence seizures recorded reported none. Overall, using a logistic regression analysis age, sex, epilepsy syndrome, or length of time of epilepsy were not significant predictors of under‐reporting. However, the use of zonisamide (*P* = 0.03) or valproate (*P* = 0.03) was significantly associated with under‐reporting. Using the video‐EEG telemetry data, we found that over‐reporting and under‐reporting were negatively correlated (phi coefficient = −0.73, *P* < 0.01), indicating that people who were likely to over‐report were unlikely to under‐report and vice versa.

## 5 DISCUSSION

Our results further support previous studies,^7^, ^8^ by showing that AS are highly misreported, with only 24% and 26% of seizures correctly identified in the over‐ and under‐reporting assessment groups, respectively.

Interestingly, patients either under‐reported or over‐reported their AS but rarely both. This pattern of reporting suggests that personality traits may have a role (eg, hypo‐ or hyper‐vigilance) or that there is some intrinsic difference between the nature of the absence seizures (however, we were unable to detect any effect of epilepsy type). Another factor is the impact of medication. Both zonisamide and valproate, antiseizure medications that have been reported to affect attention,^9^ were significantly associated with under‐reporting. This was a small, retrospective study, so caution needs to be exercised in the interpretation of these results, but they do suggest that medication, perhaps through an impact on attention, could influence seizure reporting. Although we found no significant impact of environment (inpatient VET versus outpatient ambulatory EEG) on over‐reporting, we cannot exclude over‐attentiveness due to the patient or parent/caregiver insecurity in missing possible seizures and, conversely, lack of environmental clues in the video‐EEG telemetry unit (absences may be noticed more during periods of active engagement) could have contributed to under‐reporting.

Nevertheless, our findings support the observations from other studies that there is considerable misreporting of absence seizures. This can have profound medical and social impacts, for example, over‐treatment of people who are seizure‐free, or driving whilst still having unrecognized absence seizures. Moreover, many patients had continued absence seizures despite polytherapy, and this along with the potential cognitive impact of absence epilepsy^10^ underlines that epilepsies with typical absences are not always “benign” conditions.

## CONFLICT OF INTEREST

None of the authors has any conflict of interest to disclose.

## ETHICAL PUBLICATION STATEMENT

We confirm that we have read the Journal's position on issues involved in ethical publication and affirm that this report is consistent with those guidelines.

## Supporting information


Table S1
