# Supplementary material for: How accurately do adult patients report their absence seizures?
Source: Epilepsia Open. 2023 Mar 28;8(2):641–4. doi: 10.1002/epi4.12689 (PMC10235562; doi:10.1002/epi4.12689)
Supplement: Supplementary file 1 — Table S1 [file EPI4-8-641-s001.docx]

**Supplementary Table 1: Regular medications used by patients, grouped by target**

| Drug Target | Regular antiseizure medications | Number |
| --- | --- | --- |
| SV2A | Brivaracetam | 1 |
| SV2A | Levetiracetam | 10 |
| Sodium channel | Carbamazepine* | 3 |
| Sodium channel | Eslicarbazepine* | 2 |
| Sodium channel | Lacosamide | 6 |
| Sodium channel | Lamotrigine | 17 |
| Sodium channel | Oxcarbazepine* | 3 |
| Multiple | Topiramate | 4 |
| Multiple | Zonisamide | 14 |
| Multiple | Valproate | 14 |
| Benzodiazepine | Clobazam | 8 |
| Benzodiazepine | Clonazepam | 8 |
| Benzodiazepine | Nitrazepam | 1 |
| AMPA receptors | Perampanel | 6 |
| α_2_δ subunit | Pregabalin* | 2 |
| T-type calcium channel | Ethosuximide | 3 |

*antiseizure medications usually contraindicated in absence seizures
